# Towards realist-informed ripple effects mapping (RREM): positioning the approach

**DOI:** 10.1186/s12874-024-02371-7

**Published:** 2024-10-30

**Authors:** Kevin Harris, James Nobles, Louis Ryan, Christoph Szedlak, Hannah Taylor, Rowena Hawkins, Alice Cline, Elizabeth Smith, Amelia Hall

**Affiliations:** 1 Hartpury University, Hartpury, Gloucester, UK; 2 Leeds Beckett University, Leeds, UK; 3 Sheffield Hallam University, Sheffield, UK; 4 Active Essex, Chelmsford, UK; 5 Public Health Wales, Wales, UK

**Keywords:** Realist, RREM, Causal connections, Ripple Effects Mapping

## Abstract

**Background:**

Evaluation approaches such as ripple effects mapping (REM) and realist evaluation have emerged as popular methodologies to evidence impact, and the processes of change within public health as part of whole systems approaches. Despite the various examples of their implementation across different evaluation settings, there has been little or no evidence of how they might be effective when combined.

**Methods:**

With REM’s potential to pragmatically illustrate impact, and realist evaluation’s strength to identify how and why impacts emerge, this paper develops a rationale and process for their amalgamation. Following this, we outline a realist-informed ripple effects mapping (RREM) protocol drawing upon a physical activity based case study in Essex that may be suitable for application within evaluation settings in a range of public health, whole system and physical activity settings.

**Discussion:**

Combining these two approaches has the potential to more effectively illuminate the impacts that we see within public health and whole system approaches and initiatives. What is more, given the complexity often imbued within these approaches and initiatives, they hold capability for also capturing the causal mechanisms that explain these impacts.

**Conclusions:**

It is our conclusion that when combined, this novel approach may help to inspire future research as well as more effective evaluation of public health and whole system approaches. This is crucial if we are to foster a culture for learning, refinement and reflection.

## Background

Seldom do programmes, policies, interventions or whole systems approaches (whole of systems approaches being cross sector collaborations to address social / structural challenges at a combination of levels [1], work exactly as intended within specific contexts. For those involved in evaluating these initiatives and approaches, this unpredictability poses a significant challenge given the multiplicity of impacts occurring, the channels by which these impacts come about, and the circumstances in which they happen. Simultaneously, those who invest expect certain levels of accountability and evidence for what happens.

In this paper, we provide a rationale and protocol for bringing the two approaches of realist evaluation and ripple effects mapping together to form realist-informed ripple effects mapping (RREM). Throughout this paper, we position this rationale and protocol within the following aims and objectives: 1. justification for merging the two approaches, 2. illustrating the process (the protocol) for carrying out RREM, and 3. providing insights and discussion for what this integration means and possible future directions. To strengthen these three aims and the protocol, we provide a supporting case study (see Appendix) that is utilising a RREM approach to evaluate a whole systems asset-based community development approach within a physical activity organisation in the UK. In essence, the central premise of our paper is one that calls for pragmatic innovation in evaluation practice that draws upon the strengths of merging different evaluation approaches (such as REM and realist evaluation). This is opposed to being grappled in methodological and philosophical debates about the tensions in their convergence (however we do acknowledge that there should always be critical space to discuss differences in methodological foundations). This stance is supported by Barbrook-Johnson et al., (9), who have called for more practical methods of evaluation that could aid the exploration of complexity. And in doing so, they highlight the following point;

- “Complexity-appropriate evaluation methods do not have to be sophisticated or highly technical. Often the best strategy is to co-produce and customise together with users and stakeholders a combination or hybrid of existing methods, that are adaptable, iterative and appropriate. Many methods can be repurposed for complex policies and contexts. The innovation is getting them in the right place, in the right hands, and using them in the right combination, at the right time” ([9]: pg.13).

### REM

REM is a qualitative method of capturing the wider intended and unintended impacts of an initiative. The participants of a REM workshop include stakeholders who are representative of a whole system approach or initiative. To be specific these people may be paid staff (senior and / or operational front line) representing an organisation as well as volunteers from the voluntary and charitable sector within a specific community. In addition, they may comprise of other community members (often referred to as beneficiaries of initiatives). In essence those who participate in REM are and should be part of the whole system driving the initiative under evaluation.

These stakeholders are invited to one or more participatory workshops. The workshops aim to create a visual depiction of the activities that have occurred within a given timeframe, the impacts that have occurred, and then the secondary or tertiary impacts which followed thereafter (i.e., the ripple effects). Importantly, REM focuses on understanding the interconnections between these activities and impacts.

There are several different approaches to conducting REM, as outlined in the field guide by Chazdon et al. [10], from web mapping to in-depth rippling, to theming and rippling. Nobles et al. [5] recently advanced the in-depth rippling technique by both using REM concurrent to the implementation of an initiative (rather than post-implementation) and placing more attention on the temporal nature of the activities and impacts (through use of a timeline). This version of REM has gained significant traction over the last 2–3 years in the public health context – e.g. physical activity [6, 11, 12], obesity prevention [13], health promotion [7], sexual health [14], and workplace health [15].

### Realist evaluation

Realist evaluation makes use of qualitative and quantitative methods to understand how, why and for whom something works [16] and has been applied in various health orientated and physical activity settings [4, 8, 34]. Realist evaluation is rooted in, and informed by, the philosophical realms of realism; in short, realist evaluators accept that there is a reality out there independent of our knowledge of it [16, 17]. To understand how and why changes occur, evaluators need to make sense of the generative causation at play in context; by this we mean the hidden mechanisms of change [18] that influence outcomes that will vary across different settings (contexts) [19]. Indeed, these contextual nuances will influence the outcomes we see.

Realist evaluation is usually underpinned by three interdependent phases. The first phase centres around the development of programme theories (i.e. explanatory statements) to hypothesise how, for example an initiative works, often referred to as contexts, mechanism reasoning, mechanism resources and outcomes (see 18). The second phase involves the testing and exploration of these theories via mixed methods data collection [20]. The final phase focuses on the refinement of the theories following analysis to better describe how and why the initiative works as it does.

### Bringing the ‘Real’ into REM

Both REM and realist evaluation have unique strengths. REM is a pragmatic method, which can actively engage and captivate a wide range of stakeholders. Data collection is relatively simple and can be adapted based on the resources available [12]. As a result, REM can quickly build a high-level account of what has occurred within an initiative or whole systems approach over a period of time. REM does not however surface the generative causation which is so vitally important in understanding whole system approaches and how they operate within real world systems. Conversely, realist evaluation can explore what the key mechanisms are for generating change [3], enabling us to explain how and why impacts emerge. However, unlike REM one of the challenges facing realist evaluation is the degree of stakeholder accessibility and participation; sometimes attributed to the philosophical complexity of the approach [21–23], and the conceptual jargon often underpinning it [24]. Thus, there is a strong justification for drawing on the strengths of each approach, (REM and realist evaluation), and bringing these together to form RREM.

Research suggests that both REM and realist evaluation are approaches that are open to methodological innovation and wider exploration. Realist evaluation has already seen a wide range of scholars experiment and explore the fusion of the approach with other approaches such as Q-Methodology [25–28], soft systems approaches [29] and economic evaluation [30]. This places us in a position of openness and innovation to further enhance these valuable evaluation methodologies. In Table 1 below we position REM and realist evaluation next to each other identifying some of the strengths and limitations of the approaches. The idea of convergence seeks to show the complementary nature of the two for strengthening the approaches and mitigating some of the limitations.

**Table 1:** Aligning the principles of REM and realist evaluation

Principles of REM	Principles of Realist Evaluation
Qualitative method that can allow us to capture the impacts of an initiative or whole systems approach over time	Theory driven approach that can encompass mixed methods to understand the generative causality of the impacts
Identifies the ‘what’. is going on	Explores the how and why behind the ‘what’
Participatory and engaging process for stakeholders	Potential for stakeholder accessibility and participation
Straight forward data collection method	Philosophically complex approach. Data collection could be cumbersome based on various methods used
Can capture changes over time with stakeholders	May capture generative changes over time sensitive to different contexts, but may limit engagement with wider stakeholders
Towards Convergence: RREM	
• An iterative evaluation approach, applicable for use in whole of system approaches • An approach that brings complexity driven principles into pragmatic and participatory settings • Accessible and engaging for stakeholders, using a participatory method of collecting data • Key focus on identifying, investigating and learning from impacts as a result of a programme, policies or intervention • Opportunity to develop ‘causal connections’ within and behind impact pathways • Variety of outputs including visual maps, qualitative and quantitative data	

## Methods—the RREM protocol

**Fig. 1:**
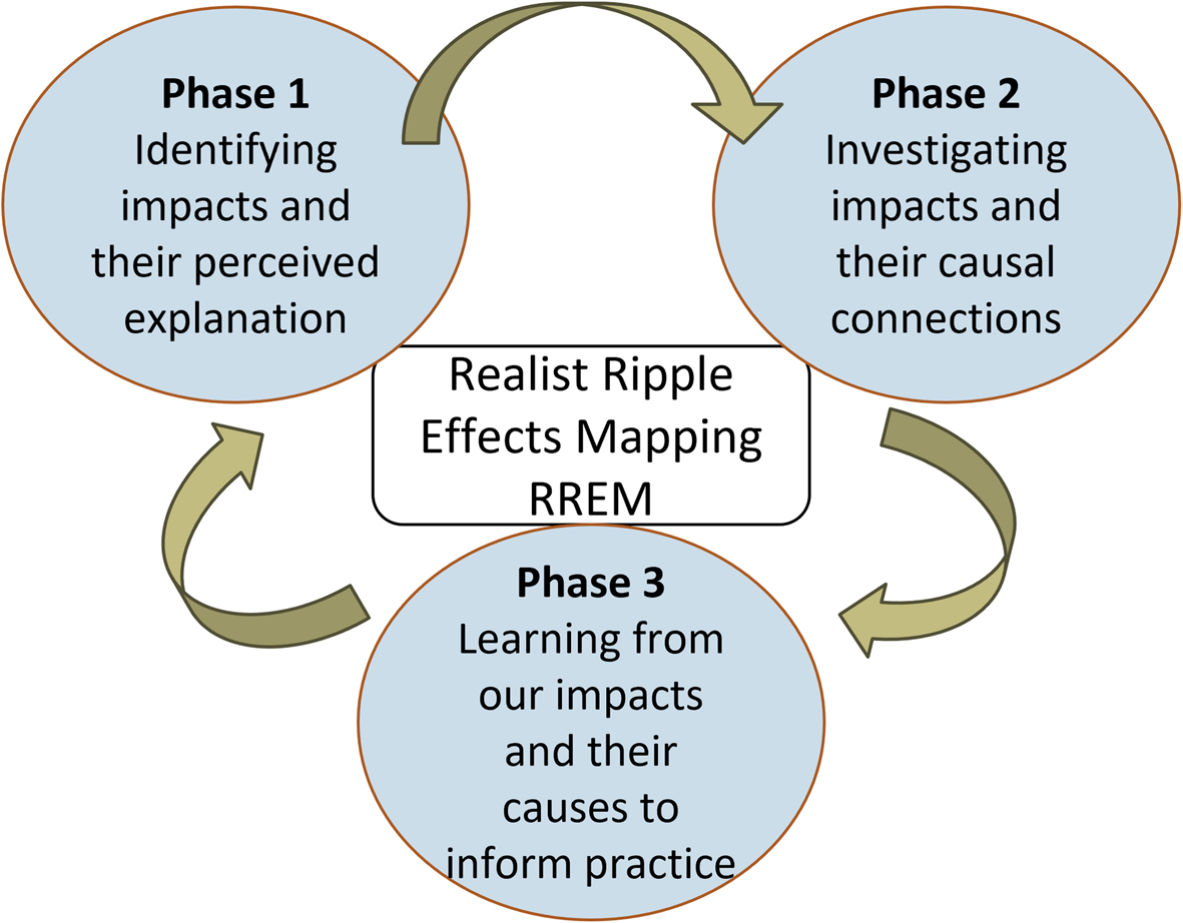
RREM approach and phases

From the outset we refer to this protocol as a RREM methodology. We present an iterative evaluation methodology that brings together the key strengths of the two approaches, which can be applied to any setting where it is important to understand impacts and the explanations of change that lead to them in whole system approaches. The RREM methodology does not favour REM or realist evaluation as the dominant methodology, and what we present is a synthesis of the two (see Fig. 1). As a starting point, we situate the traditional process of REM (for us, we refer to the Nobles et al. [5] application of REM) into a realist evaluation framework which commonly comprises the phases of developing programme theory, testing programme theory and refining programme theory. For greater accessibility, we re-frame these phases as:

- Phase 1. Identifying impacts and their perceived explanation.
- Phase 2. Investigating impacts and their causal connections.
- Phase 3. Learning from our impacts and their causes to inform practice.

The protocol that we present below is designed to suit a wide range of public health / whole system approaches and / or initiatives. Additionally, there is no prescription in regard to how long the RREM implementation process lasts. The length of each phase should be determined relative to the context of the evaluation and its aims and objectives. Within this paper we have produced an appendix that details a RREM evaluation (across the three phases) that the authors were engaged in within Active Essex (A physical activity organisation in the UK) spanning twelve months. Whilst drawing upon some examples in the paper, we provide this as a supplementary resource to this paper that may bring to life the reality of the process across the three phases.

### Phase 1: identifying impacts and their perceived explanation

**Fig. 2:**
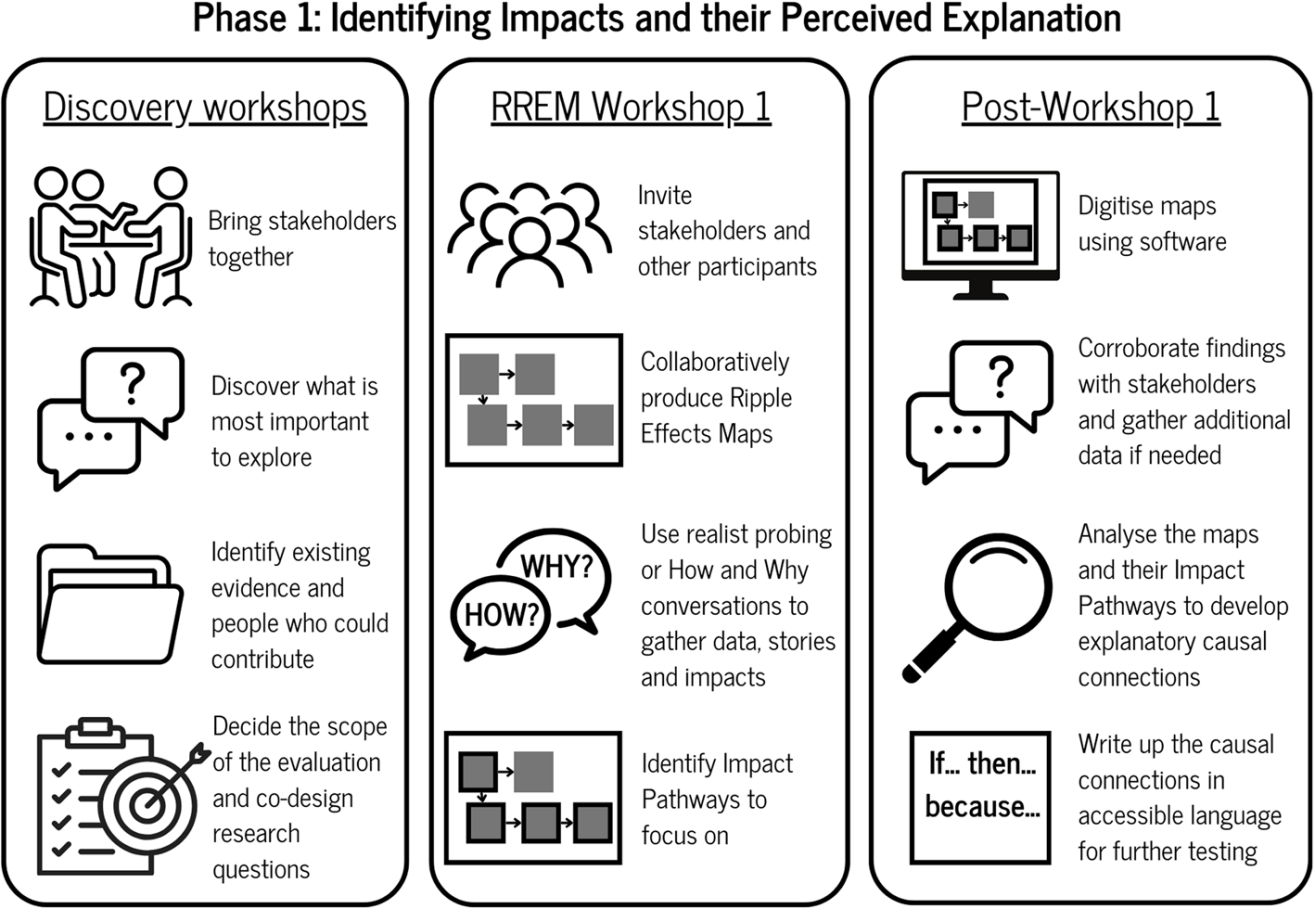
Phase one of RREM

In the first phase of RREM (see Fig. 2), the foundational stages of a realist evaluation are brought to life where the evaluation team works with key stakeholders who are engaged in a whole system approach or initiative to identify the underlying theories and explanations behind the outcomes and impacts they believe they are seeing in their work. Here, we use outcomes and impacts interchangeably on the basis that they capture the key results and contributions that are seen within a programme or system. Secondly, we deploy these key principles within the participatory and inclusive nature of the REM process [5] to encourage co-production.

#### Discovery workshops

This phase begins with ‘Discovery workshops’ that bring together stakeholders involved in, or affected by, the whole system approach or initiative. The purpose of this workshop is to confirm the aims and objectives of the evaluation, which helps to prevent stakeholders and researchers moving too quickly into the active evaluation without having clear questions, objectives, and boundaries. Discovery workshops enable the evaluation team to establish the focus of the inquiry, by asking:

1. What is important to stakeholders, and what do they think is important for exploration? For example, if looking at a broad topic, are there specific areas that should be looked at and where may there be impacts emerging?
2. Is there motivation for collaboration [31] to encourage stakeholders to take agency in the evaluation? This may involve developing mutual professional relationships [31].
3. What evidence is currently available to inform what is evaluated? Identification of these sources may highlight issues that have already been understood which may avoid duplication.
4. What is feasible and achievable for the evaluation? This requires open and honest conversations about what is practical and realistic whilst remaining focused on the aims and objectives of the evaluation.

In keeping with the participatory nature of REM the discovery workshop is important for involving a range of stakeholders invested in this work, including those who represent communities and the whole system approach under exploration. This process can be conducted in person or virtually using software such as Miro, Microsoft PowerPoint, Canva, or Vensim to visually illustrate the key themes/topics identified as important by the group. Within the discovery workshops, participants are asked to consider how and why these areas of focus are important because impacts and outcomes will naturally emerge in discussion, which the evaluation team can make note of for future exploration.

#### RREM 1: mapping provisional impacts and identifying theories to test

The first RREM workshop aims to identify impacts associated with the delivery of the initiative. The workshop follows the general principles as presented in Nobles et al., [5]. Typically, a workshop will last 2.5 to 3 h and can be hosted either in person or online with as many as 15–20 participants working on multiple maps. Here, participants should be split into groups that can contribute to the relative maps, confirmed at the discovery workshop, and be given the opportunity to tell their story, discussing the successes and challenges experienced or observed within the area of focus. This can be supported by the facilitator by using questions influenced by appreciative inquiry [5].

The workshop then encourages participants to map activities (or actions), impacts and ripple effects (i.e., those that occur because of another impact). The links between activities and impacts are key in identifying where impacts happen as a result of other impacts and are referred to as ‘impact pathways’ (multiple impacts that are linked together within the RREM workshops), see Nobles et al., [5]. However, what is different within the RREM approach is that participants are encouraged to dig deeper into the links identified between impacts. Facilitators prompt participants to explore the links by asking ‘how?’, ‘why?’ and ‘for whom?’ to understand the causality underpinning the link between activities and impacts, and what it is about context that influences these. This happens in real time during the workshop and is captured to inform the next stage of the process. Before the workshop is concluded, it is important for participants to highlight the most and least significant impact pathways that should be considered for further testing.

#### Post RREM workshop 1

After the workshop, it is useful to digitise the maps produced in the workshop utilising the software previously highlighted. As part of this production, it may be necessary to add to the map through the process of corroboration by gathering additional data from key stakeholders who were not able to attend (in addition to those who attended). These may be supported by one-to-one ‘How and Why’ conversations (inspired by Manzano’s [32] paper on crafting realist interviews) that start to uncover how and why the observed impacts and changes are occurring. This process helps to establish the impact pathways identified within the workshop, and provides opportunity for further exploration, which we discuss in the next section.

#### Developing causal connections

Having identified the impact pathways to focus on, the research team develop ‘causal connections’ that illustrate the deeper causality underpinning these impact pathways. These causal connections represent the realist causal impact pathways that within a realist ontology [33], sit behind and beneath the traditional REM impact pathways. These causal connections are developed using realist heuristics such as context mechanism resource, mechanism reasoning and outcomes [18], or explanatory statements to uncover the generative causality behind how and why impacts are generated. Using a retroductive approach that theorises and tests hidden mechanisms [33], researchers are able to use their inferred knowledge and understanding (from the discovery workshop, RREM workshop 1 and grey literature) of the evaluation topic to make explicit the key reasons about how and why an impact has or has not occurred. This process of causal connectivity is particularly useful for ‘what impacts do we expect to see in the upcoming months’ part of the mapping (usually, the next three months is proposed as a suitable time period [5]. In our application of RREM, our causal connections are drawn up through the explanatory process of “IF… THEN… BECAUSE…” statements. This format has been chosen to ensure that the causal connections are digestible for all participants to avoid disengaging people with realist jargon (please see Appendix for specific example).

At this stage, the causal connections identified could be linked to literature to identify potential underpinning theories (see Greenhalgh and Papoutsi [35]) that represent the area of focus (see Appendix for example). Overall, this whole process culminates in the production of a RREM map that is underpinned with initial causal connections ready for testing and exploration in phase 2.

At the end of the RREM phase 1, the following actions will have taken place:

• Discovery workshops
• RREM workshop 1
• Digitising map and sharing with participants
• Identifying causal connections

### Phase 2: investigating impacts and their causal connections

**Fig. 3:**
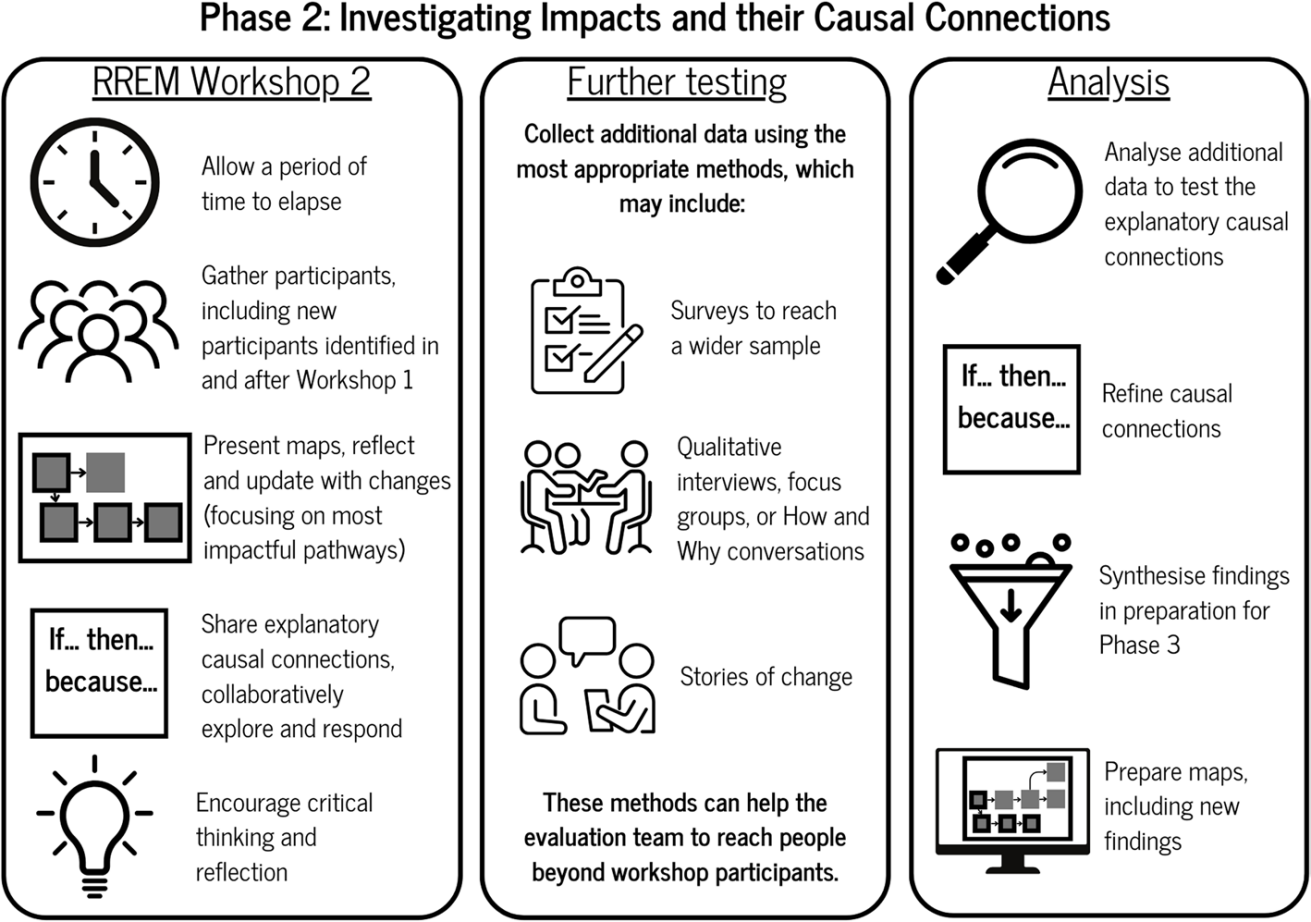
Phase 2 of RREM

Phase 2 (see Fig. 3) involves the explicit investigation of the impact pathways and causal connections that were identified in phase 1. Within the realist approach, this is a testing and exploratory phase using data from RREM workshop one and two alongside a variety of additional qualitative and quantitative methods to explore how, why and for whom these pathways are manifesting.

#### RREM 2 workshop: testing and refining impact pathways and causal connections

Prior to delivering the second RREM workshop, a period of time (usually a three-month gap [5] is recommended to encourage further activities and impacts to occur (however, this gap could be longer, or even shorter). This is also important to allow the ‘what impacts do we foresee happening in the coming months’ to take shape. In essence, the space between workshop 1 and workshop 2 provides the whole system approach or initiative with time to ‘live’ and ‘breathe’ within its unique context. After the appropriate amount of time has elapsed, participants are gathered together once again for the second RREM workshop.

Within the second workshop (and as depicted within the phase 2 graphic, see Fig. 3), the completed maps from phase 1 are presented to participants with the intention to create participation, inclusion and critical engagement. The most significant impact pathways are also illustrated and explained at this stage. In addition, the causal connections that lie beneath, within and around the impact pathways are also explained to participants. For many in attendance at this workshop, it will be their first sight of the maps, and in particular, the causal connections that have been developed. It is here where they have the opportunity to reflect upon the map, make any changes to what is being presented and to refine accordingly.

Following on, participants reflect upon what has happened over the last three months making changes to the impact pathways and discussing the causal connections that were developed as part of a testing process Participants have the freedom to critically discuss these causal connections, which involves the process of adapting and refining them ready for the next iteration of RREM. This process also includes the introduction of rival causal connections or theories, which further encourage the participants to respond in more depth. This is a unique realist contribution to the RREM process in that theories are being tested in a collaborative manner. This is seldom achieved within a realist evaluation as participants do not always have an explicit role across the three stages of developing, testing and refining theories. We would argue that within a traditional realist evaluation participants are involved in developing programme theories yet their role in testing and refining is more passive and implicit. On the contrary, within RREM, both the participants and the research team co-produce the process of theory testing and refinement explicitly in inclusive and collaborative settings.

The role of the research team in this workshop is to stimulate evaluative consciousness, using questioning influenced by the identified causal connections, wherein participants start to think critically about their activities and impacts [31], and consider the strengths and weaknesses of what has occurred. In addition and in accordance with a knowledge exchange environment [36, 37], participants are also bringing their own critical offerings to this learning environment which in turn fosters learning within the research team. The key output from this workshop is an adapted REM map that shows development, growth, and reflexivity in regard to the impact pathways and respective causal connections.

#### Further testing and investigation of impact pathways and causal connections

After the RREM 2 workshop, an additional phase of testing and exploration takes place. Here, the research team operationalise a series of realist-informed qualitative and quantitative methods to further test the impact pathways and causal connections to explore how, why and for whom the pathways apply. In accordance with realist evaluation, a methods neutral approach is taken [20] where those methods that are most suited to testing the impact pathways and causal connections are selected. These methods may consist of surveys that provide more extensive reach to test the impacts on a wider sample. For example, in a community development setting, this may be valuable in terms of accessing key members who did not attend the mapping workshop but are still given an opportunity to inform the development of the maps. The same can also be said for implementing qualitative methods of interviews and focus groups [32]. In particular, being able to have deeper ‘How and Why’ conversations with people who may have, or not, been at the workshops helps to further investigate the impact pathways and accompanying causal connections. There is also the potential here to experiment with other qualitative methods focusing on vignettes or stories of change [38] that represent and bring to life some of these impacts.

For example, as depicted in more detail within the case study (see Appendix) a key impact pathway emerging centred upon how important training in strengths based community development and engagement (what we refer to as ABCD in the case study) helped staff within a leisure facility setting in receipt of the training make better connections with the community around them. These connections and relationships led to (for example) ripple effects of staff developing skills in understanding, connecting and building relationships with communities more. Having developed more explanatory causal connections (if, then, because statements) about this pathway, we then utilised stories of change to go deeper into what this impact pathway really meant for staff into what it was about the training in ABCD that enabled them to relate to their community more (see Appendix).

Essentially, this further investigation is the explicit testing of the impact pathways and causal connections that will be shaped for presentation and sense making in the third phase of RREM. This additional wave of testing helps to bring more value and credibility to workshop findings that might not always reflect the true reality of what is happening in practice. This use of additional and complementary methods has been advocated for by REM methodologists [12].

#### Analysing and synthesising findings

Having mobilised the selected methods to support the testing, this stage of phase 2 involves the research team familiarising themselves with the data, and then moving deeper into analysis. As Nobles et al., [12] state, there is no prescribed approach for how impact pathways are analysed in REM. The analytic approach may adopt an inductive or deductive [10, 12] content analysis that draws upon the data presented in the maps. However, within a RREM approach there are multiple sources of data that require analysis and integration that draw upon the maps as well as additional qualitative and quantitative methods. It is here where we suggest that a realist inspired retroductive position [33] is drawn upon, whereby the research team searches for generative causal insights that lie beneath the surface of what can actually be seen. For example, the CMMO [4, 18] heuristic may assist with coding contexts, reasoning and reactions that are at play within the impact pathways and connections under test. This iterative process of data analysis and integration results in the provisional refinements to the RREM map and respective impact pathways and their causal connections. This process culminates in a rich data set that informs the illustration of a refined set of impact pathways and underlying causal connections ready for presentation, deliberation and learning for phase 3.

At the end of the RREM phase 2 the research team will have fulfilled the following tasks:

1. Prepared and delivered RREM workshop two.
2. Used RREM workshop 2 to present impact pathways and supporting causal connections to participants for amendment and refinement.
3. Mobilised a series of additional methods for testing post workshop 2.
4. Analysed data and synthesised findings for presentation in phase 3.

### Phase 3: learning from our impacts and their causes to inform practice

**Fig. 4:**
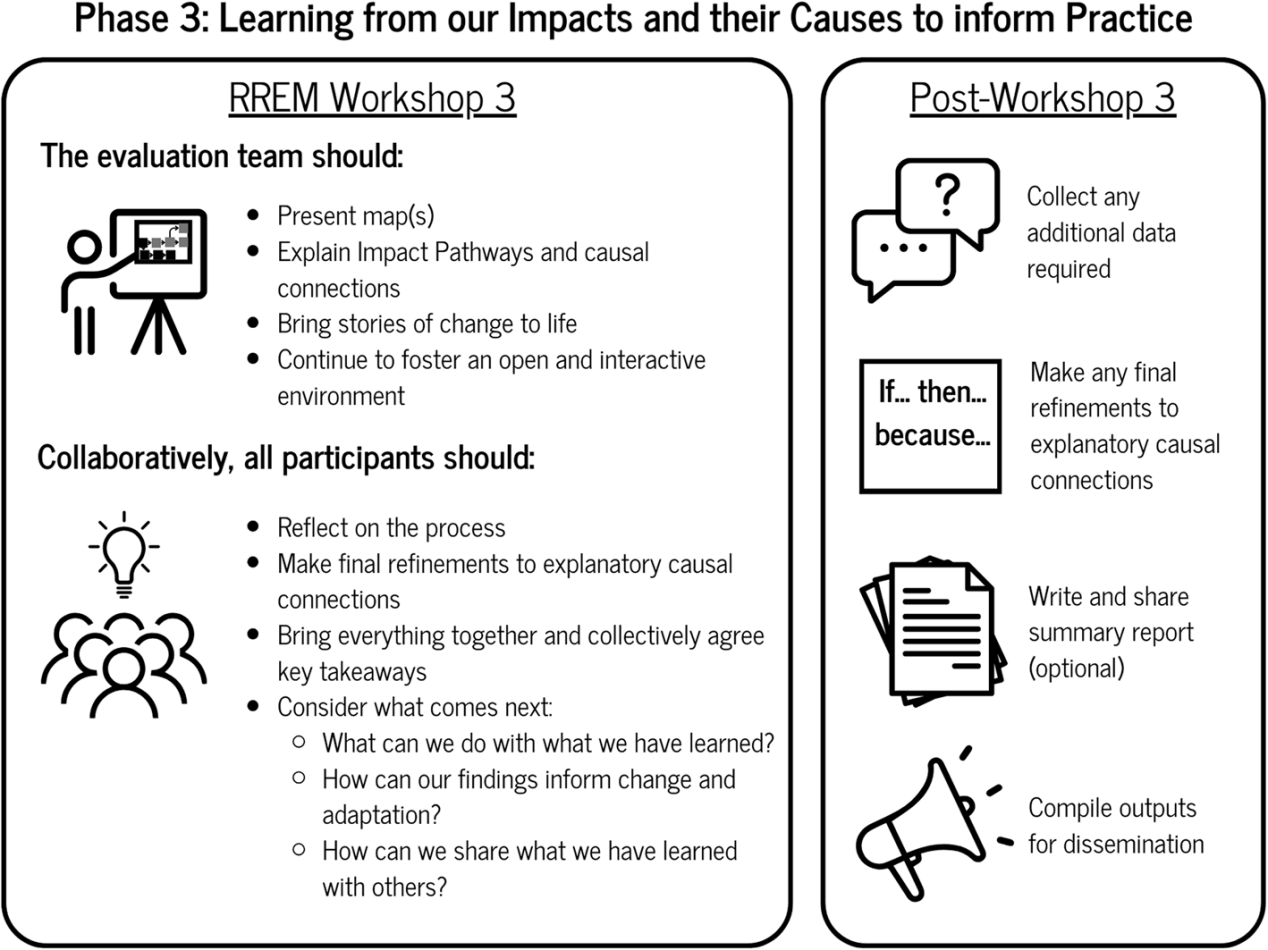
Phase 3 of RREM

As we move into the third and final phase of RREM (see Fig. 4), by this point an additional period of time has elapsed to take into account the testing of the theories alongside the additional methods of exploration. In line with phases one and two, there is no firm prescription on the duration of time between phase two and three which should be guided by the aims and objectives of the evaluation, and the resources available. In this final phase of the RREM process the research team operationalise key stages of learning and refinement. This idea of learning and refinement is an important point of alignment for REM and realist evaluation because both approaches encourage this endeavour. For example, REM promotes the appreciative inquiry approach that encourages participants within a co-design environment to ‘discover, define, dream and design’ approaches that have had a positive impact [39]. And, within a realist position, the programme theory refinement stage encourages stakeholders to make sense of the theories that have been tested, and what their refinements look like. In turn, Shulha et al., [31] suggests that whole system approach or initiative stakeholders consider what these refinements mean for informing use and action in initiative design.

#### RREM workshop 3: presenting and refining learning

The final workshop aims to present findings that represent the refined causal connections and impact pathways. In terms of participation, it is of value to involve all stakeholders consistently across the phases in each workshop, while also establishing routes or engagement with stakeholders who are unable to attend all of them. In practical terms, the research team brings to life a near-finalised RREM map which also includes suggested refinements to the impact pathways and causal connections. At this stage the research team has also the opportunity to present crafted “stories of change” [38] to illustrate the impact pathways and causal connections identified (refer to Appendix for an example). In this workshop, rather than accepting them as a given, these findings are presented in a provisional sense that allow for corroboration on the part of the participants to discuss and refine what is presented in the maps. Below, we list some of the questions that guide this workshop:

• What are the impacts that remain valid and what are the explanations behind them?
• What are the suggested causal connection refinements and what role do participants have in inclusively agreeing them and shaping them?
• Can agreement be established on what the refined map looks like?
  • Where do we take the findings and the map next?
• How does, and how can this process inform future changes or adaptations and who needs to know?

A key point is that this stage should be one where consensus on the refined impact pathways and casual connections should be mutually agreed. To allow this collaborative element to take shape, the research team implemented the appreciative inquiry principles of discover, define, dream and design [39]. Such a process then enables the participants to make further changes to the map (if required) and then consider what happens next.

#### Fostering use and making learning actionable for the future

The time following this workshop is crucial for those commissioning RREM to consider the findings. Instead of seeing the findings as a summative end, participants and/or stakeholders should use this stage as an opportunity to reflect, act, or plan to act on the findings to foster ‘use’ [31]. As Shulha et al., [31] and Cousins [40] state, there is little point in evaluation, if the findings are not put into action. This may lead to initiative modifications, as well as identifying new areas of focus which have emerged through the preceding processes. What is more, identifying new areas and impacts of focus creates a rationale for continuing the RREM process in a new iteration, on the basis that knowledge is fallible and there should be no end to assessing the merit and worth of provision. This sparks synergy with the realist cycle [16] for developing, testing and refining theories again once an initial iteration is complete. Overall, such an approach helps to develop a continuous developmental cycle [41] of learning that runs alongside the development of a whole system approach or initiative. By the end of the RREM process, participants will have an idea of what impacts have occurred, how they have evolved over time, and what the explanation is behind them. This creates an opportunity to take ownership over this evidence and consider what this means for action and dissemination.

At the end of the RREM phase 3 the RREM community will have fulfilled the following tasks:

1. Prepared refined maps and respective impact pathways and connections ready for presentation in workshop 3.
2. Delivered workshop 3 and presented refined map(s).
3. Provided an environment of deliberation and consensus to agree on the maps using appreciative inquiry principles.
4. Produced a refined map supported by causal connections. This map may be supplemented with or embedded within an overall report summarising the three phases with the supporting data captured in phase 2 as well as stories of change.
5. Compiled a set of outputs that are ready for dissemination and action for future iterations of RREM and initiative design.

## Discussion

Having introduced the RREM protocol, in this section of the paper we consider the implications of the RREM method for practice and future direction. We will cover the following areas; *practical considerations*, *challenges*, and *future directions* for progressing the approach.

### Practical considerations

#### Staff resource requirements

Given that this RREM process involves the combination of two traditionally discrete methods, it is important to identify how a RREM project is appropriately resourced. This considers the role of the researchers and supporting people within the RREM team as to who is involved in what phase, and subsequent stage of RREM. Within the practical delivery of the workshops, where feasible, it is important to have a facilitator allocated to capturing information for the REM output. Specifically, there will be different orientations of this role where there will be a facilitator (or facilitators) charged with the responsibility for capturing the content that relates to the impact pathways, but also another role in beginning to shape the causal insights that represent the realist position of this work. This essentially means that facilitators need to have a skill set that is not only conducive with REM but also one that is familiar with realist evaluation. This dual facilitation will enable participants to discuss what has occurred within their work from the level of identifying impact, and then to the deeper level of understanding the how and the why. The facilitator should also guide the conversations and seek to obtain a greater level of detail. Furthermore, outside of the practical delivery of the workshops, evaluation teams need to consider who is responsible for the ‘desk based’ tasks of data analysis as well as the additional ‘realist’ orientated fieldwork data that needs to be captured. Having multi-disciplinary teams assembled who are embracing different methodological domains is fundamentally important.

#### Participant recruitment

Consideration should be given to how and when participants will be recruited to take place in the RREM process. Evaluation teams will need to decide who is responsible for providing access to participant samples and data that is required for the evaluation, ideally within the discovery phase of the RREM cycle. However, this is something that needs to be managed and monitored throughout all three RREM phases. Reflection on the recruitment and engagement of participants may also be required, for example are those who are participating likely to have a bias towards the initiative (i.e., are you likely to engage participants who have had a negative experience?). It is also important to consider who the whole system approach or initiative intended to reach compared to those taking part in the evaluation.

#### Challenges

Although the RREM protocol presented here provides a novel method of evaluation, we must acknowledge that there are several challenges that may need to be overcome. The challenges we illustrate here are not exhaustive, but in accordance with our Essex case study bring to life some of our own experiences in mobilising the approach.

For example, engaging with the RREM method requires the evaluator(s) to straddle the different dimensions and philosophies of both realism and pragmatism. There is the additional challenge of navigating the varied perspectives of stakeholders, commissioners and practitioners and their often different expectations for future impact and outcome from RREM. This was very much an experience for us in relation to this case study where for some, RREM simply provided a mechanism to legitimise their work [42] and showcase the positive aspects of what was being mapped. We would encourage future RREM methodologists to acknowledge this potential motivation for collaboration of RREM being used to evidence ‘what works’ [43] as opposed to being used to explore causal connections that demonstrate the deeper causality underpinning the impact pathways, which may uncover negative or challenging aspects of people’s work.

The RREM method relies on wide active participation engagement from stakeholders, users, and facilitators. Barriers to participation may include time, cost, resources, lack of knowledge, training, and understanding of the importance of the process. Therefore, establishing a clear motivation for collaboration [31] from the outset is important for understanding why people want to be engaged and what is driving their participation. Without knowing this there is danger of stakeholders becoming what Nichols et al. [44] refers to as subjugated, (as in not feeling part of the process). In our application of the case study we have been able to mitigate this through the discovery stages of RREM and further testing of impact pathways with wider stakeholders. This has enabled us to capture a wider sample of stakeholders engaged in the area under investigation beyond traditional REM workshops. However, we also acknowledge that deeper reflection and sense making is needed to check and challenge whether this challenge is being confronted.

Thus, establishing meaningful inter-professional relationships between the evaluation team and stakeholders [31, 36] is a key factor in keeping people motivated and engaged throughout so that there is value in the process. We should also not ignore that the RREM process also requires an evaluation team that is large enough and that is capable of completing all phases to provide a meaningful evaluation. In the example of this case study we are fortunate to have a robust multi-disciplinary research team that has brought together academic methodologists alongside embedded researchers within Essex. Nevertheless we have had to carefully manage the processes of data collection, travel to specific sites and afford appropriate time to data analysis (of essentially multiple methods).

### Future directions

As outlined, RREM brings together principles of both REM and realist evaluation. RREM addresses the limited stakeholder accessibility that sometimes can be identified with realist evaluation, while building on the participatory and accessible nature of REM. In doing so, there is the potential for evaluators to consolidate their technical findings into plain-speaking language, which benefits not only the key stakeholders involved with the work but means that the findings can be presented to both practitioners and policy-makers, helping to bridge the gap between research and practice.

As we move forward, the next steps are to experiment with the RREM approach exploring the value it brings to the field of evaluation practice. We encourage researchers, evaluators, and wider stakeholders to apply this approach and to reflect on its use for evaluation practice. Although we outlined a model for this approach, we encourage future research to further develop RREM when applying it to individual contexts. For example, we have demonstrated that there is no time limit to how RREM should be applied and it is up to research and evaluation teams to decide what is sufficient and achievable in this respect. There is also nothing to oppose RREM approaches that perhaps only focus on discrete phases of the RREM cycle. For example, if simply implementing phase 1, it can be a valuable tool for developing causal connections and helping to design whole system approaches and initiatives in a participatory way. The same can also be said for the other phases which in their own right may offer value to a specific focus of an evaluation.

And finally, the flexibility incorporated in RREM provides an exciting and novel area for future research. We believe that an encouraging environment currently exists for exploring the utility of different complexity driven and pragmatist evaluation approaches [9] and we should seize this moment.

## Conclusion

This paper has presented the novel merging of realist evaluation and REM to create a method which is suitable for understanding what happened, and why, with regards to public health whole of system approaches and initiatives. Through the RREM protocol, presented across the three phases, we believe that those seeking to understand the impact of their work will have a more in depth understanding of causal connections underpinning the impact pathways identified. And, they have the opportunity to do this through a highly applied, collaborative, and participatory process. We acknowledge the challenges of RREM and encourage evaluation designers to consider what is possible based on the available resources that are needed to operationalise the approach. This consideration should run alongside the level of motivation for collaboration [31] that key stakeholders have as they enter into the approach. For example, why are they participating and what is it that they wish to benefit from in a practical and or transformational evaluation sense [45]?

However, despite these challenges and practical considerations, the synthesis of realist and REM principles offers significant promise for advancing evaluation practice. Thanks to the advancement, and advocacy of complexity informed approaches to evaluation [3] there is a strong rationale for evaluation approaches such as realist evaluation being implemented in complex settings. Yet, how these complexity and theory driven approaches are mobilised in practice with wider stakeholders is open for advancement in the way that they become more accessible, meaningful and inclusive for people. And it is at this point where the pragmatic focus of REM offers value for demystifying the challenges presented by theory driven approaches such as realist evaluation. Moreover, aligning the realist approach with REM promotes this combination of hybrid methods that involves stakeholders. To reiterate a central point of this paper, the REM approach offers value for engaging people, stimulating interest which then lays the foundation for exploring causal and explanatory insight into the inner workings of programmes, policies and interventions. Nevertheless, a suitable point to end this paper echoes Barbrook-Johnson et al., (9, pg 14) advice, that: “the choice we face is to not whether to do a ‘complex’ or a ‘simple’ evaluation, but whether to do an effective or ineffective one”, and this is where RREM has the opportunity to evolve. This is premised in the spirit and openness of exploring the use and combination of different evaluation approaches advocating methodological innovation [2].

## Appendix

### RREM case study—Active Essex

#### Introduction

In 2023, Hartpury university was commissioned to conduct a realist-informed ripple effects mapping evaluation for Active Essex, an active partnership overseeing sport and physical activity in Essex, England. The evaluation focused on the impact of Asset Based Community Development (ABCD) training across Essex, within a whole systems approach. ABCD training provision, coordinated by Active Essex, is a whole systems approach as it seeks to address the barriers to people being physically active within Essex, with the training provided across sectors (third sector, local authority, statutory services) to influence the system to work collaboratively to tackle the multiple, interrelated factors (social and structural) that influence physical activity levels. This, in essence involved building the capacity and capability of staff through ABCD principles which meant engaging more collaboratively with communities in a strengths based way to take more control over the assets in their community that may tackle physical inactivity. This case study provides an example of the evaluation in practice to support the protocol outlined in the paper. Active Essex commissioned the evaluation team to explore the impact that ABCD had with those who received the training, coordinated by Active Essex. The training was provided for locally trusted organisations (LTOs) such as third sector organisations. For Active Essex to identify the impact of the training amongst over 250 training recipients, it was felt ripple effects mapping was an important way to map out the impacts, with realist evaluation used concurrently to understand the ‘how?’ and ‘why?’ explaining the impacts.

### Phase 1- identifying impacts and their perceived explanation

#### Discovery workshop

To help confirm the parameters of the evaluation, the discovery workshop played an important role in engaging with stakeholders and identifying the focus of the evaluation. 8 stakeholders were identified as key to the discovery workshop, as well as 2 members of the evaluation team. These stakeholders held roles that included implementing ABCD within their job role but to also support LTOs in implementing ABCD. The workshop was held online and asked the participants to answer questions using to establish the focus of inquiry in the evaluation. Using Miro, participants gave feedback to 3 questions:

1. What is important?
2. What evidence do we have?
3. What can we focus on?

Questions were asked in this order to encourage participants to narrow their focus for the evaluation. The evaluation team used this as an opportunity to ask participants ‘how?’ and ‘why?’ to explore the causality behind their responses. The discovery workshop was also used to identify those who should be invited to future RREM workshops.

#### RREM workshop 1

The first RREM workshop was hosted in person, facilitated by the evaluation team. The workshop began with the evaluation team introducing the focus for the workshop, as well as walking through the process for participating in a ripple effects mapping workshop (see Nobles et al. [5]). The participants were encouraged to ‘warm up’ for the workshop through having conversations with other participants about their involvement in ABCD, with appreciative inquiry questions used to influence conversation.

1. Can you share some positive examples of ABCD have you been involved in?
2. What unique skill sets or assets do you have in your community?
3. What impact have you had on your community or area?

Throughout the process, the evaluation team would engage in conversations, asking questions to encourage the participants to share more of the reasoning and detail behind their responses. This was followed by the mapping exercise where participants were split in groups relative to their locality in Essex so that the map was more specific to their involvement. A member of the evaluation team facilitated a map each to encourage conversations around the topic to map out the impacts. Participants were in free-flowing conversation, with the evaluation team often writing down the impacts for the participants while they shared their experiences. The workshop structure is outlined within Table 2 with the evaluation team facilitating the whole process. To ensure that the workshop retains its realist nature, the evaluation team must continue to ask questions of participants to understand the ‘how?’ and ‘why?’ of the impacts being mapped.

**Table 2:** Example of PREM workshop structure

Activity	Prompts to help participants
*Introduction and warm up*	
The facilitator(s) should introduce the RREM workshops to the participants and initiate the warm up activity. The activity can be innovative in a way that encourages conversation and reflection, using questions influenced by appreciative inquiry, although it is vital to consider challenges and things that have not gone well	• What is important to you? • What is important to your organisation? • What motivated you to contribute to the area of focus? • What would be the ideal outcome of your project? • What have the positive outcomes been? • What have been some of the challenges? • What partnerships played an important role?
*Mapping activity*	
Mapping the impacts The facilitator(s) works with participants to confirm a timeline for the mapping. The participants will then map the impacts they identify and link them to other impacts	• What are the impacts that participants have observed or experienced? • Were there any additional impacts as a result? • Focus on the impacts • Estimate the time if you are not sure • It is fine to work forwards or backwards from an impact
2. Adding extra detail Participants are encouraged to add additional detail that provides additional context. This can also explore the causal connections between the impacts	• Can you highlight what impacts were unintended? • Can you identify who has been impacted and why? Were these intended or unintended? • Were there any financial impacts? • Are there wider contextual factors that are important to these impacts? • What do you predict will happen in the next 3 months?
3. Most and least significant impact pathways Participants should select pathways based on the perceived impact in relation to the time and resource committed to the pathway. The researcher should use this opportunity to dig deeper with questioning	• Can you highlight the 2 most significant impact pathways in your view? • Can you highlight the 2 least significant impact pathways based on the resource and time invested into the pathway? • Why have you selected these pathways?
*Reflections and next steps*	
The facilitator(s) should use this opportunity to allow participants to reflect and share thoughts from their participation. The facilitator(s) should also share the next steps so the participants are clear and able to ask any questions	• What are your reflections from the workshop? • Were there any impacts that surprised you? Can you tell us why?

### Post workshop 1

**Fig. 5:**
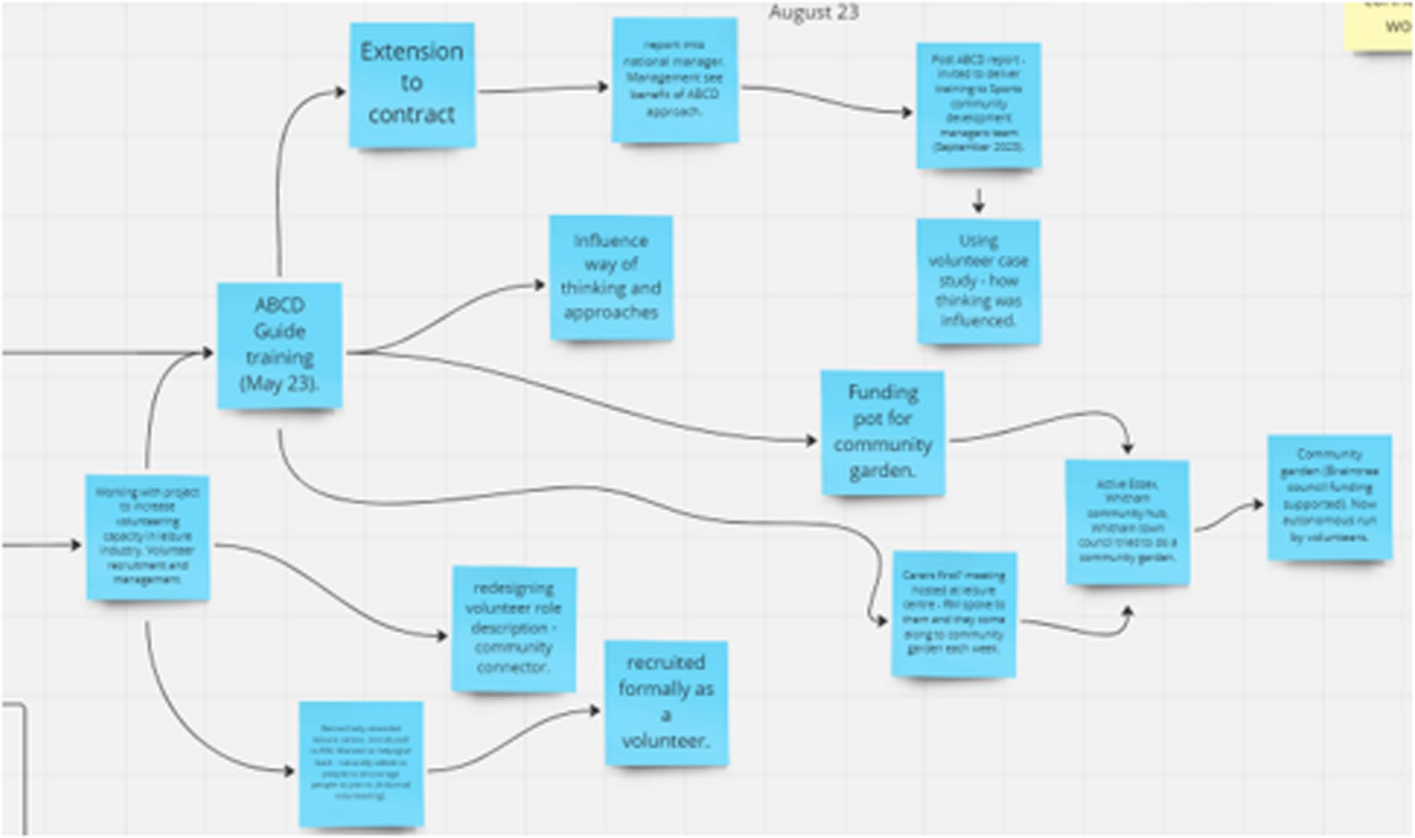
Snapshot of map

First, we digitised the map. During this process, which requires full immersion of the researcher in the data collected in workshop one, we identified impact pathways. Figure 5 provides a snapshot of the map focusing on one specific impact pathway. The impact pathway focused on how individualised ABCD training of staff in a leisure centre impacted on the community engagement and community mobilisation.

#### Causal connections

Following on, we identified causal connections within the impact pathways. We then identified two theories that underpinned these causal connections: the transformational and servanthood leadership theories. Overall, we developed six casual connections. Below are two examples. The first is based on the transformational leadership theory element called “individual consideration” (e.g., Bass & Riggio [46]), where leaders (Active Essex) display consideration for the unique needs and desires of each LTO/stakeholder (leisure centre).

- IF the leader (Active Essex) considers the unique needs and desires of each LTO and stakeholder, THEN the LTOs and stakeholders feel mutual accountability BECAUSE they feel appreciated and important in achieving a worthwhile cause and therefore believe they are working together to achieve similar aims.

The second incorporates humility and stewardship (e.g., Van Dierendonck [47]), which is elementary to the servanthood leadership theory.

- IF Active Essex prioritises humility, working for the LTOs and stakeholders and providing stewardship, THEN this approach facilitates the growth of “followers into effective leaders” reducing the need for Active Essex as LTOs become more self-reliant and joint up directly with the system BECAUSE Active Essex have provided tailored training, resource and support for the system and stakeholders/LTOs to become joined up.

### RREM phase 2: investigating impacts and their causal connections

#### Workshop 2

We invited the stakeholders back to reflect on the identified impact pathways and causal connections. Post workshop one and during the analysis phase, we also identified additional stakeholders that could provide more specific and detailed information aspects of the impact pathways that were unclear. This enabled us to gain a much deeper understanding of the individual contexts and also enabled us to refine the causal connections. The questioning during the workshops was informed by the causal connections identified. This means, we would ask how stakeholders might feel appreciated, how they became self-reliant, and the process that led to this. As a result we developed and identified Jack’s Story of Change, which explores the process of how ABCD training for the manager of the leisure centre enabled community mobilisation:

ABCD training must be context specific, I was able to take this and get rid of the jargon and apply this to my context. I mean people around here do not know what “assets” means, but ask them what they are good at, that works. You could say I was lucky, my organisation was well set up for the ABCD approach and also that is the way I work. It [ABCD training] made me quite motivated and reassured, and gave me the confidence to move forward. It is definitely a bottom up approach. You have to start on the ground and you need some freedom and autonomy to move forward. I had this and my manager was in total support, you do not often have this [organisational support] because when you do relational community work results are not instantaneous. Trust is important. … My main aim was to increase the volunteer capacity, which is a difficult task, yet, when you focus on their abilities, their likes, their passions, suddenly it can snowball. It becomes more of a connection through the volunteers to the community. Take this for example, we had two volunteers who were interested in developing a community garden. They did this all by themselves, I just helped to get some funding, now they have a lot of the community helping them and even the local council is interested in how they set it up. They [the local council] tried but have failed so far. So, this ABCD approach has certainly something to offer. (Story is summarised from interview data).In this example, we can see the area of humility and stewardship being displayed as well as the collaborative working environment, which is based on mutual accountability and common goals.

#### Post workshop2

##### Further testing and investigation of impact pathways and causal connections

Similar to post workshop one, we identified areas where we need to collect additional data, this would focus on specific aspects of the causal connections. This included individual interviews, focus groups, as well as surveys aimed to more clearly define our causal connections.

**Fig. 6:**
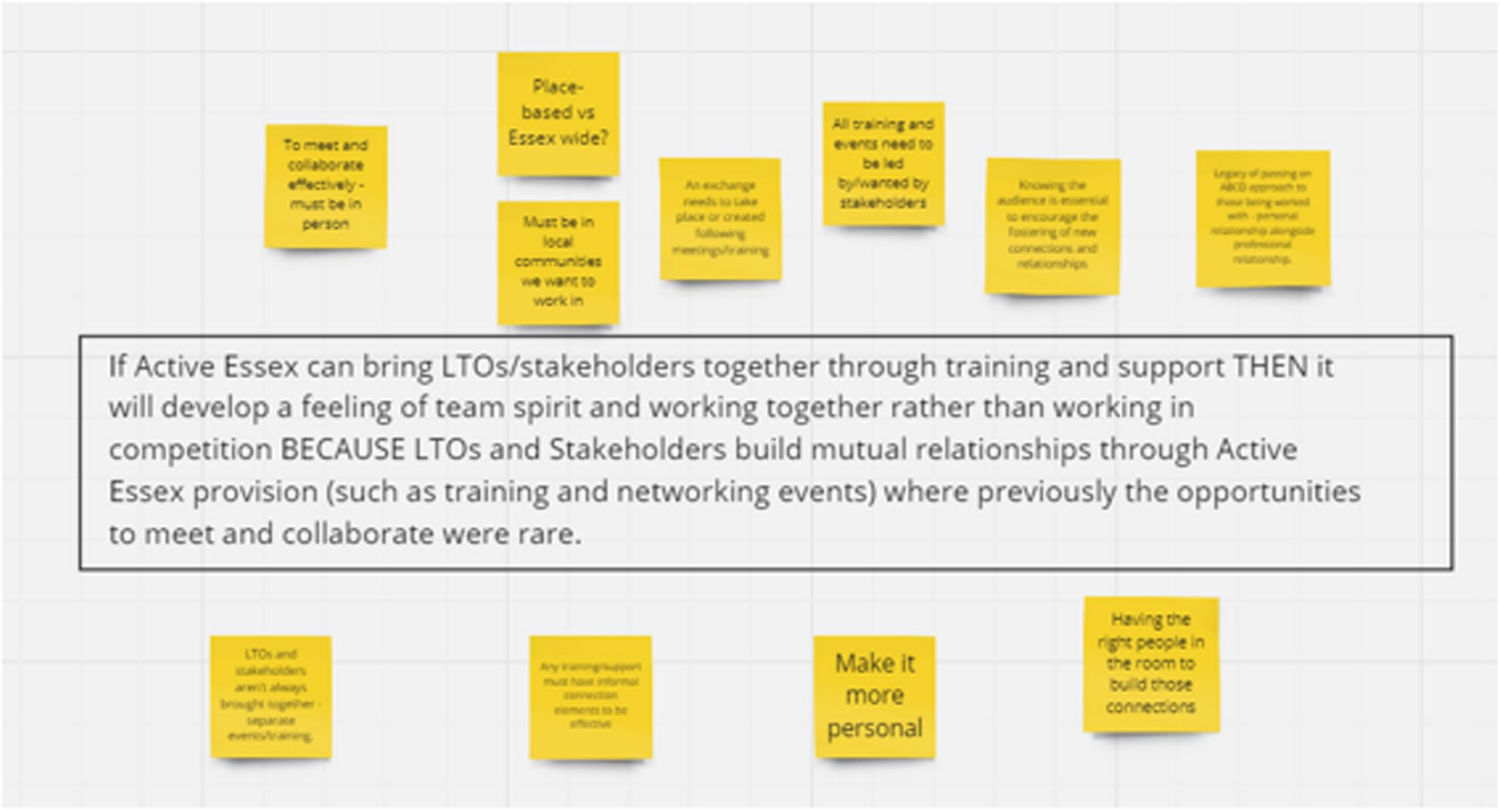
Causal connection

For example, during a focus group, which included ABCD guides, we asked for their thoughts on the following causal connection (see Fig. 6), which reflects “Inspirational Motivation”, the transformational leadership element stimulating team spirit.

##### Analysing and synthesising findings

The team adopted a retroductive position (see Jagosh [33] for further reading) when analysing the data. This allows the evaluation team to explore the mechanisms that may or may not trigger in different contexts and lead to expected or unexpected outcomes. This is part of the rigorous testing process to further refine the causal connections, an iterative process where the evaluation team had to be open to changing the causal connections as more data and insight is gathered. As a result of this exercise (Fig. 6), we replaced the word “mutual relationships” with “personal relationship”, to highlight the importance of building strong and collaborative relationships.

### RREM Phase 3: learning from our impacts and their causes to inform practice

#### Workshop 3: presenting and refining learning

Within this phase, the evaluation team worked with stakeholders and participants to help share the learnings and what this may mean for their practice. The workshop allowed the participants to come together and reflect on the map and the refined causal connections. Where the map and causal connections were new to some participants, it gave them an opportunity to add further detail; however this workshop had greater focus on the causal connections.

In the first part of the workshop, participants were presented with the latest version of the map to add any final impacts and connections, as well as corroborating the highlighted impact pathways in the map (the stories of change were also presented in this process). This was followed by presenting the causal connections to the participants, asking them to check and challenge to ensure that any additional insight was captured, and the causal connections had gone through thorough refinement. This was important for the evaluation team to collect feedback on the current iteration of the causal connections and to ask questions for further explanatory thinking behind the feedback. Depending on the level of refinement and congruence between evaluators and participants, phase 2 and phase 3 could be repeated until data saturation.

##### Fostering use and making learning actionable for the future

Post workshop, the map and the causal connections were in effect *‘handed’* over to the stakeholders of the initiative. In keeping with a collaborative approach, this was done with opportunities to explore the findings so that they could be put into action. The evaluation team hosted a workshop with stakeholders to ‘unpack’ the findings to understand the generative causation behind the change that did or did not occur as a result of this whole systems approach. This considered how Active Essex would apply their learning into future whole systems approaches whilst translating findings into accessible formats so participants of the RREM process could also learn from the findings.

## Data Availability

No datasets were generated or analysed during the current study.
